# Hypothermia during Carotid Endarterectomy: A Safety Study

**DOI:** 10.1371/journal.pone.0152658

**Published:** 2016-04-08

**Authors:** Serena Candela, Raffaele Dito, Barbara Casolla, Emanuele Silvestri, Giuliano Sette, Federico Filippi, Maurizio Taurino, Domitilla Brancadoro, Francesco Orzi

**Affiliations:** 1 NESMOS (Neurosciences Mental Health and Sensory Organs) Department, School of Medicine and Psychology, Sapienza University, Sant'Andrea Hospital, Rome, Italy; 2 Cardiothoracic Vascular Sciences Department, School of Medicine and Psychology, Sapienza University, Sant'Andrea Hospital, Rome, Italy; 3 Surgical Anesthetic Sciences Department; School of Medicine and Psychology, Sapienza University, Sant'Andrea Hospital, Rome, Italy; Sapienza University of Rome, ITALY

## Abstract

**Background:**

CEA is associated with peri-operative risk of brain ischemia, due both to emboli production caused by manipulation of the plaque and to potentially noxious reduction of cerebral blood flow by carotid clamping. Mild hypothermia (34–35°C) is probably the most effective approach to protect brain from ischemic insult. It is therefore a substantial hypothesis that hypothermia lowers the risk of ischemic brain damage potentially associated with CEA. Purpose of the study is to test whether systemic endovascular cooling to a target of 34.5–35°C, initiated before and maintained during CEA, is feasible and safe.

**Methods:**

The study was carried out in 7 consecutive patients referred to the Vascular Surgery Unit and judged eligible for CEA. Cooling was initiated 60–90 min before CEA, by endovascular approach (Zoll system). The target temperature was maintained during CEA, followed by passive, controlled rewarming (0.4°C/h). The whole procedure was carried out under anesthesia.

**Results:**

All the patients enrolled had no adverse events. Two patients exhibited a transient bradycardia (heart rate 30 beats/min). There were no significant differences in the clinical status, laboratory and physiological data measured before and after CEA.

**Conclusions:**

Systemic cooling to 34.5–35.0°C, initiated before and maintained during carotid clamping, is feasible and safe.

**Trial Registration:**

ClinicalTrials.gov NCT02629653

## Introduction

Carotid artery diseases cause stroke in 10–20% of the cases [1]. Carotid Endarterectomy (CEA) is of proven efficacy to reduce the risk of stroke and TIA [2]. The efficacy had been shown many years ago by the North American Symptomatic Carotid Endarterectomy Trial (NASCET) and Asymptomatic Carotid Atherosclerosis Surgery (ACAS) studies. Both studies proved the long-term benefit of CEA to prevent cerebrovascular diseases, both in symptomatic and asymptomatic patients.

CEA is associated with peri-operative risk, due both to emboli production caused by manipulation of the plaque and to the potentially noxious reduction of cerebral blood flow by carotid clamping [3]. In the NASCET study [4, 5] peri-operative strokes occurred in 5.5% (non-disabling 3.7% and disabling 1.8%), and death in 1.1% of the enrolled patients. In the ACAS study [6] the cumulative risk of peri-operative stroke or death occurred in 1,5% of the patients. While recent data confirm the entity of the risk [7], improved medical therapy and prevention have probably reduced the hazard from carotid stenosis. Present indication criteria for CEA or stenting may, therefore, need a critical appraisal, which reflects the new balance between pros and cons of the carotid surgery. There is indeed growing consensus for conservative approaches, especially in patients with asymptomatic carotid artery disease [8]. Thus, while a growing body of evidence supports the role of CEA in stroke prevention, physicians should undertake every possible effort to maximize the efficacy of CEA by reducing the peri-operative risk.

Mild hypothermia (34–35°C) is probably the most effective neuroprotective approach [9]. Hypothermia, by reducing brain energy metabolism [10], decreases the metabolic needs in conditions of reduced supply, such as it occurs during hypoperfusion [11, 12]. In addiction hypothermia unwinds a number of mechanisms involved in the progression of the tissue damage following ischemic event [13]. Most of the supportive data were obtained in animal models of ischemia [14]. For instance, hypothermia reduces the release of excitotoxic neurotransmitters and the production of free radicals, and inhibits pro-inflammatory and apoptotic pathways [15, 16, 17, 18]. Hypothermia modifies immediate early gene expression [19] and reduces aquaporin expression and edema [20].

Several phase II trials have shown the safety and feasibility of cooling subjects with stroke, in the hours following onset of symptoms. Early interventions show the highest benefit.

Because the potential damage caused by CEA is of ischemic nature, it is a consistent hypothesis that hypothermia substantially reduces the risk associated with the surgical procedure. Aim of our study was to determine whether systemic cooling to a target temperature of 34.5–35°C initiated before, and maintained during CEA, is feasible and safe.

## Materials and Methods

### Study population

**Fig 1:**
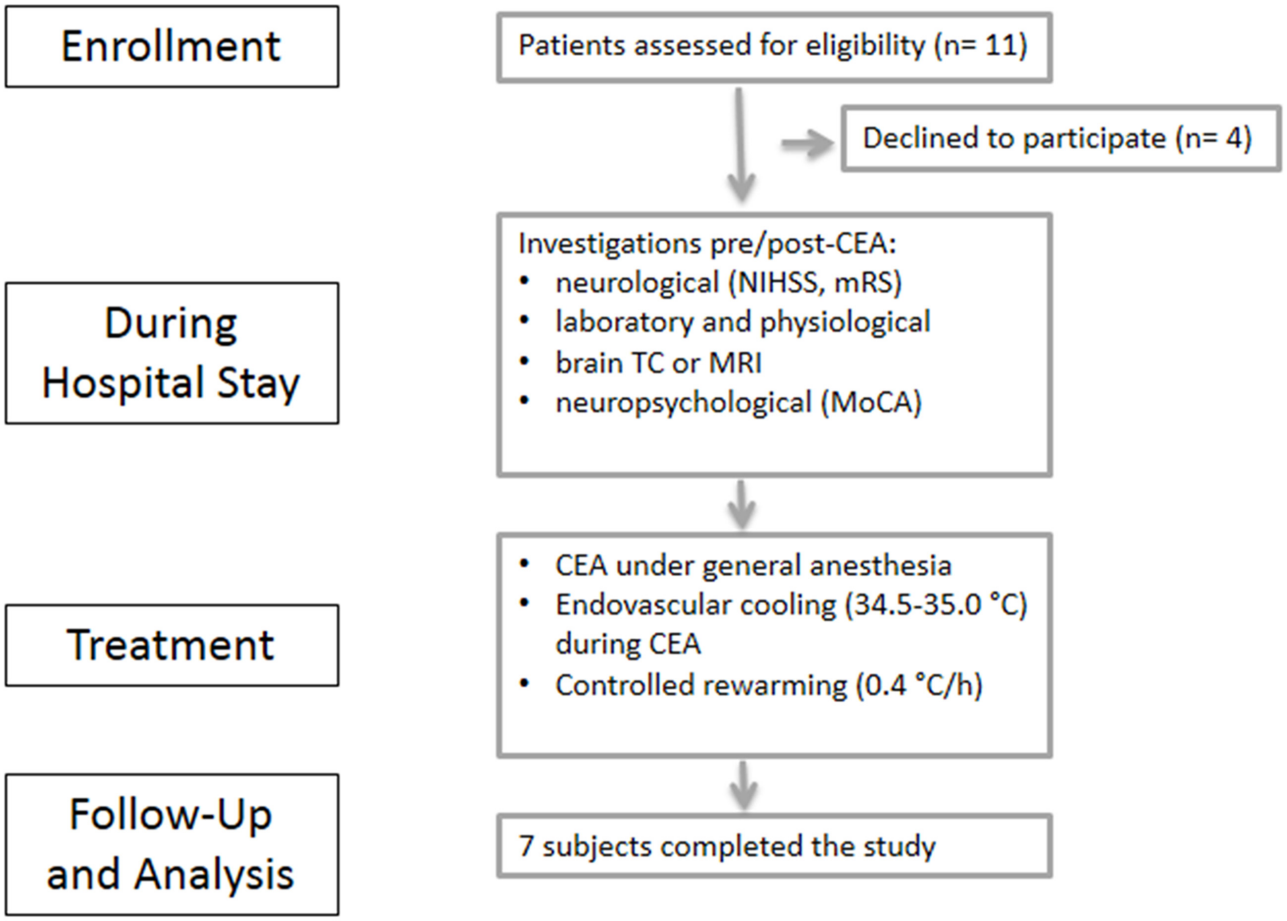
Flow chart of the study progression.

Consecutive patients referred to the Vascular Surgery Unit for CEA from April 2013 to June 2015 were included in the study. Fig 1 shows the study flow diagram. Inclusion criteria were: eligibility for CEA as established by independent vascular surgeons, low (<4) Anesthesia Risk Assessment (ASA) and age ≥ 18 years. Exclusion criteria were: evidence (as a result of peri-operative neuroimaging or of any pre-inclusion investigations) of intracranial hemorrhage, tumor, encephalitis, or acute brain focal lesion; progression or instability of neurological status; conditions that may be exacerbated by hypothermia, such as hematological dyscrasias, oral anticoagulant treatment with INR ≥ 1.7; severe pulmonary disease; severe heart failure (defined as a New York Heart Association (NYHA) score of III or IV); history of myocardial infarction within the previous 3 months; angina pectoris in the previous 3 months; severe infection with a C-reactive protein > 50 mg/dl, or a clinical diagnosis of sepsis; blood oxygen saturation below 94%, allowing a maximum of 2 L/min oxygen delivered nasally to achieve this; bradycardia (<40 beats/min); use of a monoamine oxidase inhibitor such as selegiline in the previous 14 days; severe hepatic dysfunction; severe renal dysfunction; pregnancy; other serious illness that may confound treatment assessment or increase the risks of cooling; social or other conditions that according to the investigator’s judgement might be a major problem for follow-up. In addition women of childbearing potential were excluded unless a negative test for pregnancy had been obtained prior to inclusion. All patients were investigated for common cardiovascular risk factors, included hypertension (defined as systolic blood pressure (BP) ≥ 140 mmHg, diastolic BP ≥ 90, or current use of antihypertensive medications) and abnormal laboratory findings.

### Assessments

Neurology evaluations included stroke severity (NIHSS score) and dependency (mRS> 2), which were assessed at the following time points: admission, awakening from anesthesia, discharge from the hospital, and 30 days after CEA. A critical care stroke neurologist performed all examinations.

Laboratory data were collected on admission and one day after CEA. The laboratory data included cholesterol (total, LDL, HDL cholesterol and triglycerides), leukocyte count, platelet count, antithrombin III, Prothrombin Time (PT) and Partial Thromboplastin Time (PTT). Self-reported clinical history of diabetes, smoking or alcohol consumption was also recorded.

Physiological data were collected just before, during and the day after hypothermia. Data included heart rate, blood pressure and blood gas analysis.

Magnetic Resonance Imaging (MRI) and TC were performed before the intervention and within 30 days post CEA. Radiological outcomes included occurrence of new acute ischemic (evaluated by Diffusion Weighted Images) or hemorrhagic (including micro-hemorrhages visible on Gradient Echo-MRI) lesions. Images were evaluated by neuroradiologists blind to protocol.

Neuropsychological assessment was performed by means of Montreal Cognitive Assessment (MoCA), before and within 30 days after CEA.

The Sant’Andrea Hospital Ethics Committee approved the study (reference number 168/2013) and participants gave written informed consent. The successful completion of the feasibility phase allowed us to register the study (S2 File), on ClinicalTrials.gov, for continuation of the study and further analyses. The ClinicalTrials.gov identifier is NCT02629653. The authors confirm that all ongoing and related trials for this intervention are registered.

### Treatment

All patients underwent general anesthesia. Induction was performed with propofol and fentanyl. Muscle relaxation for tracheal intubation was induced with rocuronium bromide, and maintained by infusion of sevoflurane and remifentanyl. Hemodynamic instability was treated according to the indications of the anesthesiologist. Atropine and adrenaline were administered in case of bradycardia. Heparinization was performed before clamping the carotid artery. Anesthesia was continued, following completion of the surgical procedure, during the controlled rewarming to be gradually discontinued when the body temperature of 36.2°C was reached.

Clinical monitoring was performed by trans cranial Doppler (TCD) and bispectral index (BIS). Cerebral oximetry was evaluated by INVOS-4100. The temperature was measured by means of bladder and nasopharyngeal probes.

Hypothermia was obtained by means of endovascular cooling (Zoll system), to the target temperature of 34.5–35.0°C (assessed by bladder thermometer). The Zoll IVTM is an endovascular cooling system that consists of a control module (either CoolGard 3000 or Thermogard XP), a CoolGard start-up kit, and an ICY catheter (either IC-3585 AE or IC-3585). The equipment was provided and the patient’s health insurance was paid by Movi Spa (www.movigroup.com). The whole procedure was carried out during general anesthesia. The ICY catheter was inserted and cooling initiated just before the beginning of the surgical procedure. The cooling was carried on concomitantly with the surgical procedure. Clamping of the carotid was performed once the target temperature was obtained. Controlled, passive rewarming (at the rate of 0.4°C/h) was initiated just after release of the carotid clamping. For sake of prudence patients were assisted in intensive care unit during rewarming and recovery from anesthesia.

Carotid surgery was performed routinely. Briefly, following longitudinal incision along the anterior border of the sternocleidomastoid muscle the common carotid artery, the carotid bifurcation, the internal and external carotid arteries were isolated and clamped following administration of 5000 units of heparin. A longitudinal incision on the anterior surface of the carotid bulb, extending to the proximal portion of the internal carotid artery, allowed the gradual isolation and removal of the atheromatous plaque. In case of a residual dissection at the distal edge of the endarterectomy the intima was secured by means of a 7–0 polypropylene tackling suture (Kunlin suture). The arteriotomy was sutured using a Dacron patch and a 5–0 or 6–0 polypropylene suture.

### Outcome measures

The primary safety outcome was the incidence of any adverse event at 1 month. Severe adverse events were defined as any life-threatening event including pneumonia (diagnosed on the basis of clinical signs or symptoms), myocardial infarction and parenchymal hemorrhage. Non-severe safety outcomes included incidence of bradycardia (<40 beats/min), cardiac arrhythmia, hypertension, hypotension and any coagulation disorders.

## Results

A total of 11 consecutive participants were enrolled. Four patients were excluded because they declined to participate. All the 7 subjects completed the study. Table 1 shows the demographic and clinical characteristics of the patients. All the subjects presented with at least two vascular risk factors (including age >75 y). On admission all the patients presented no neurological deficits (NIHSS = 0) or disability (mRS = 0).

**Table 1:** Patient characteristics.

*ID*	*Age (years)*	*Col*	*BP*	*LLT*	*DM*	*ICS*
1	66	+	+	+	-	L
2	70	-	+	-	+	L
3	85	-	+	-	-	L
4	75	+	-	+	+	L
5	74	+	+	+	+	L
6	73	+	+	+	+	L
7	80	+	+	+	-	L

Col: cholesterol ≥ 200 mg/dl; BP: Hypertension; LLD: Lipid Lowering therapy, including statin and non-statin drugs; DM: Diabetes Mellitus; ICS: Internal Carotid Stenosis, Left or Right.

CEA was carried out successfully according to the standard procedure. Shunt was placed in 3 patients because of severe flow decrease, intended as a decrease of the peak of systolic velocity under 10 cm/sec detected in the middle cerebral artery by using TCD.

**Fig 2:**
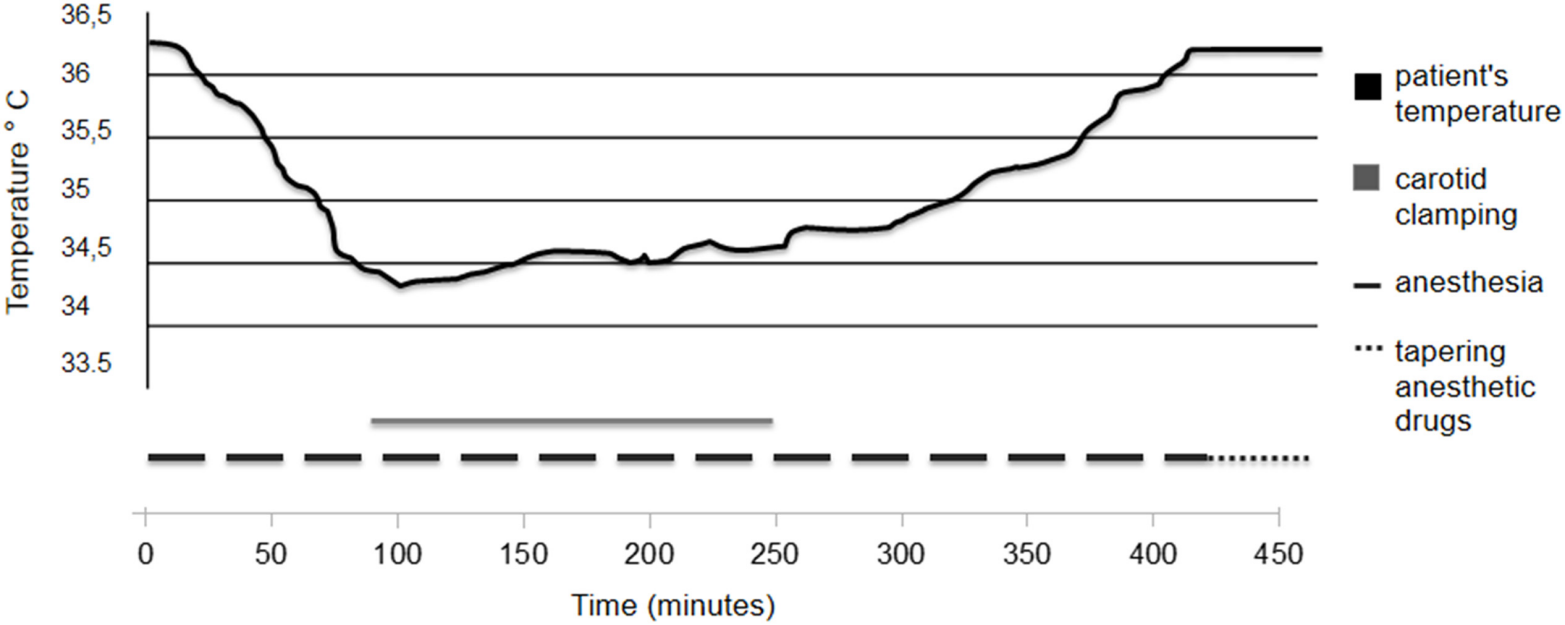
Representative time course of body temperature during and following CEA.

Time required for catheter insertion, in the right femoral vein ranged from 10 to 20 minutes. No groin hematoma was found in association with catheter insertion. Target temperature of 34.5–35.0°C was reached in 70 ± 8 minutes (Table 2) and maintained for all the duration of carotid clamping. A representative time course of the body temperature is shown in the figure (Fig 2).

**Table 2:** Hypothermia related clinical assesments.

	*before hypothermia*	*during hypothermia*
Temperature (°C)	36,1 ± 0,3	34,5 ± 0,1
Time to target temperature (min)		70 ± 8
Heart rate (per min)	73 ± 6	67 ± 13
INVOS values, L	75 ± 12	59 ± 8
INVOS values, R	79 ± 10	59 ± 7

Values are expressed as mean ± standard deviation. INVOS: percent brain tissue oxygen saturation.

Following CEA, all the subjects recovered promptly from anesthesia. There were no adverse events, with the exception of transient bradycardia occurred in 2 cases. The minimal heart rate observed was 30 beats/min. During the hypothermia the average cardiac rate was 67 ± 13 (Table 2). The null NIHSS and mRS scoring remained unchanged in all the patients, on awakening from the anesthesia, prior to discharge from the hospital and 30 days after CEA. There was, therefore, no difference in the clinical status before and after CEA.

There were no significant differences in the laboratory and physiological data measured before and after CEA (Table 3).

**Table 3:** Clinical and laboratory values.

	*pre-operative*	*post-operative*
Systolic blood pressure	130 ± 8	162 ± 35
Diastolic blood pressure	79 ± 4	73 ± 12
Platelet count	189 ± 47	154 ± 43
Antithrombin III	80 ± 4,6	78 ± 13
PT	11 ± 1	11 ± 1
PTT	31 ± 2	58 ± 49
Leukocytes	6 ± 2	7 ± 3

Values are expressed as mean ± standard deviation. Platelets and leukocytes are expressed as 10^3^/uL. Antithrombin III is expressed as percentage. PT (Prothrombin Time) and PTT (Partial Thromboplastin Time) are expressed in seconds.

TCD monitoring consistently revealed during the hypothermia a change in the shape of the middle cerebral artery wave, represented by an increase of the systolic peak and a decrease of the diastolic plateau. The phenomenon probably reflects the hypothermia-induced increased distal vasoconstriction.

Five patients performed the 30 days neuroimaging follow-up. The results showed no difference from the baseline. Two patients skipped the MRI follow up.

Subjects were dismissed from the hospital within 2 days from the surgery time, except for 2 patients because of a transient postoperative hypertension, which caused a cautious extension of the recovery time, in intensive care unit, from one to two days.

## Discussion

The results of this study suggest that systemic cooling to a target temperature of 34.5–35.0°C, initiated before and maintained during carotid clamping, is feasible and safe.

In our study the procedure was carried out during general anesthesia. Hypothermia to the target of 34–35°C *per se* does not require general anesthesia. Oral administration of buspirone and meperidine are usually sufficient to treat the discomfort and shivering consistently associated with the systemic cooling [14]. We made the choice to take advantage of the general anesthesia, required by the surgical procedure, to prevent the patient discomfort during the controlled rewarming. Thus, the anesthesia was extended to the post-operative recovery. The additional anesthesia time was of about 120 minutes.

Both rewarming and recovery from anesthesia were carried out in intensive care unit. This was a prudential approach, which the results of this study show not to be necessary. We encountered no side effects, except for two cases of bradycardia successfully treated with atropine and dopamine. Other changes observed in this study and potentially linked to the hypothermia include thrombocytopenia, increase of amylase blood content, prolonged PR and QT intervals and sinus bradycardia. All the changes were mild, not relevant from the clinical point of view and transient.

The TCD modifications observed during the hypothermia are thought to represent vasoconstriction distal to the middle cerebral artery. The phenomena is transient and apparently without any consequence.

A limit of the study is the small sample size, which results, however, congruent with ethical concern.

The study suggests that whole body cooling, to a target temperature of 34.5–35.0°C, is safe and feasible. The procedure might be considered in order to reduce the risk associated with carotid clamping during CEA.

## Supporting Information

S1 File STROBE statement checklist. (DOC)

S2 File Original study protocol. (PDF)

S3 File Relevant data set of each patient. (XLS)
