# Supplementary material for: Hypothermia during Carotid Endarterectomy: A Safety Study
Source: PLoS One. 2016 Apr 8;11(4):e0152658. doi: 10.1371/journal.pone.0152658 (PMC4825931; doi:10.1371/journal.pone.0152658)
Supplement: S2 File — (PDF) [file pone.0152658.s002.pdf]

CEACOOL

Body Cooling during carotid endarterectomy

no-profit, open, mono-centric, feasibility study.

Proponents

Brancadoro D, Candela S, Casolla B, Dito R, Filippi F, Orzi F, Sette G, Silvestri E, Taurino M
University of Rome "La Sapienza", Sant'Andrea Hospital, Rome, Italy

Rational

Carotid EndArterectomy (CEA), by removing local causes of downstream altered circulation, improves cerebral hemodynamics and provides an effective prevention of stroke and TIA. The intervention itself, however, causes immediate risk of stroke or death, and it is also an issue whether the temporary reduction of blood flow associated with clamping of the artery, during the surgical intervention, may trigger long-lasting brain tissue dysfunction.

Mild hypothermia (34.5-35 °C) is probably the most effective approach to protect the brain from ischemic insults. Most of the supportive data were obtained in animal models of ischemia. Several phase II trials have shown safety and feasibility of cooling subjects with stroke, in the hours following onset of symptoms. Early interventions show the highest benefit.

It is, therefore, a solid hypothesis that performing CEA during hypothermia substantially reduces the risk due to a potential, temporary hypoperfusion, associated with the surgical procedure.

Aim

To determine whether systemic cooling to a target temperature of 34.5-35°C initiated before, and maintained during CEA, is feasible and safe

Methods

Inclusion criteria

1. Subject eligible for CEA, without progression of symptoms, with low (<4) Anesthesia Risk Assessment
2. Age ≥ 18 years;
3. Written informed consent.

Exclusion criteria

1. Evidence from a CT or MRI scan or from other pre-inclusion investigations of an intracranial haemorrhage, a tumour, encephalitis, or any diagnosis of acute brain focal lesion;
2. Progression or instability of neurological status
3. Conditions that may be exacerbated by hypothermia, such as haematological dyscrasias, oral anticoagulant treatment with INR ≥ 1.7, severe pulmonary disease, severe heart failure (defined as a New York Heart Association (NYHA) score of III or IV), history of myocardial infarction within the previous 3 months, angina pectoris in the previous 3 months, severe infection with a C-reactive protein > 50 mg/dl, or a clinical diagnosis of sepsis;
4. Blood oxygen saturation below 94%, allowing a maximum of 2 L/min oxygen delivered nasally to achieve this;
5. Bradycardia (<40 beats/min);
6. Body weight > 120 kg;
7. Allergy to pethidine, use of a monoamine oxidase inhibitor such as selegiline in the previous 14 days, severe hepatic dysfunction, or severe renal dysfunction;
8. Pregnancy. Women of childbearing potential are excluded unless a negative test for pregnancy has been obtained prior to randomisation;
9. Other serious illness that may confound treatment assessment or increase the risks of cooling;
10. Social or other conditions that according to the investigator's judgement might be a major problem for follow-up.

Treatment

Cooling will be initiated 60-90 min before CEA by endovascular cooling (Zoll system) to the target 34-35°C (assessed by bladder thermometer). The Zoll IVTM system is an endovascular cooling system that consists of a control module (either CoolGard 3000 or Thermogard XP), a CoolGard start-up kit, an ICY catheter (either IC-3585 AE or IC-3585 CO or IC-3893 AE or IC-3893 CO), a catheter convenience kit for catheter insertion (CO models only), thermal probes and cables. All the devise components have CE mark. The Low temperature will be maintained during the CEA procedure, followed by gradual, passive, controlled rewarming (0.4 °C/h). Type of anaesthesia will be decided according to good clinical practice. The

cooling procedure will be, therefore, carried out during the anaesthesia procedure required by the surgical intervention. There is a chance that the duration of the anaesthesia be longer that required, but all the efforts will be undertaken to keep the anaesthesia time as short as if there were no cooling.

Outcome measures

Primary Outcome is the incidence of any adverse event at 1 month. Severe adverse events were defined as any life-threatening event including pneumonia (diagnosed on the basis of clinical signs or symptoms), myocardial infarction and parenchymal hemorrhage. Non-severe safety outcomes included incidence of bradycardia (<40 beats/min), cardiac arrhythmia, hypertension, hypotension and any coagulation disorders. Serious Adverse Events will be reported to Zoll Circulation Company, to Ethics Committee and to Ministry of Health.

Sample size

The trial will be carried out on no more than 10 subject. An interim analysis will be carried out after inclusion of the first 3 subjects.

Data collection

Data will be collected as following, on paper forms:

Baseline (Pre-CEA)

1. Date of birth, sex;
2. Medical history and vascular risk factors;
3. Body temperature (bladder), blood pressure, and heart rate;
4. Scores on the mRS;
5. NIHSS;
6. Laboratory tests, including full blood count, serum glucose, electrolytes, INR, PTT, aPTT, C-reactive protein, NSE;
7. Electrocardiogram;
8. MRI scan with DWI, and GRE sequences or TC;
9. Registration of MCA blood flow with transcranial Doppler (TCD) (peak systolic velocity, PSV)
10. MoCA

During CEA Procedure

1. Monitoring and registration of MCA blood flow with TCD (peak systolic velocity, PSV)
2. Systemic heparinization and monitoring with ACT test

Post-CEA

1. Scores on the mRS;

2. NIHSS;
3. MRI scan with DWI and GE sequences or TC;
4. Neurological and systemic adverse events
5. MoCA

Ethical and Administrative Aspects

1. No profit, feasibility
2. Cooling system provided by the Zoll Ciculation company
3. Cooling disposable catheter kits provided by the Zoll Circulation company
4. MRI is a routing exam
5. Analysis are included in the good clinical practice procedure for CEA
6. No additional cost for Sant'Andrea Hospital

Contact

Francesco Orzi
NESMOS Department
Neurology Unit
University of Rome "La Sapienza"
francesco.orzi@uniroma1.it
